# Leptomeningeal Carcinomatosis and Myelophthisic Anemia as Initial Manifestations of Metastatic Lobular Breast Cancer

**DOI:** 10.1155/crom/5631996

**Published:** 2025-08-26

**Authors:** Ogaga Urhie, Kally Dey, Kirtan Patolia, Shiraz Fidai, Michael Alebich

**Affiliations:** ^1^Department of Internal Medicine, John H. Stroger Jr. Hospital of Cook County, Chicago, Illinois, USA; ^2^Department of Pathology, John H. Stroger Jr. Hospital of Cook County, Chicago, Illinois, USA

## Abstract

Breast cancer can spread to the brain and bone, usually presenting as parenchymal and osteoblastic lesions, respectively. We present a unique case of a 59-year-old woman undergoing treatment for invasive lobular breast cancer who presented with nausea, vomiting, headache, and generalized weakness. Her clinical presentation and subsequent evaluation led to a discovery of leptomeningeal carcinomatosis and myelophthisic anemia presenting simultaneously as her initial metastases. She was treated with weekly paclitaxel and intrathecal methotrexate, with noted cerebrospinal fluid response. She continues to follow up with the oncology clinic.

## 1. Introduction

Breast cancer has a high propensity to metastasize to distant organs with profound multisystemic effects. Metastases, for which the evaluation is symptom-driven, may occur at any point during treatment. Treatment should account for features of the metastatic site and further control of the underlying cancer.

## 2. Case Presentation

A 59-year-old woman with a history of estrogen/progesterone receptor positive (ER/PR+) and human epidermal growth factor receptor 2 negative (HER2−) invasive lobular carcinoma (ILC) of the breast, previously treated with tamoxifen, presented with 2 weeks of immediate postprandial vomiting and generalized weakness. Vomiting was becoming more frequent, with constant nausea. She developed a new occipital headache soon after the onset of vomiting. The headache occurred at different times of the day, preventing her from falling asleep and waking her from sleep. She had not tried any medication for the headache. She suffered a motor vehicle accident (MVA) 3 months prior that caused bilateral hearing loss that had recently gotten worse. She did not have fevers, chills, diarrhea, abdominal pain, or tinnitus. At the time of our initial examination, she was having nausea, headache, blurry vision, and dizziness.

**Figure 1:**
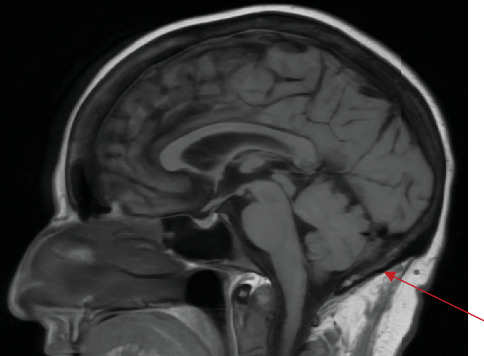
Sagittal MRI showing posterior cerebellar leptomeningeal enhancement.

Her laboratory workup on admission is presented in Table 1. CA15-3 and CA27-29 were obtained due to suspicion of increased disease activity and were found to be significantly elevated. Computed tomography (CT) chest, abdomen, and pelvis showed sclerotic bones and mild pleural thickening. A nuclear medicine scan, obtained due to a report of a prior abnormal brain magnetic resonance imaging (MRI), showed osteoblastic metastatic change in the xiphoid and degenerative findings in the bones seen on CT imaging. A brain MRI showed a suggestion of subtly symmetric leptomeningeal enhancement along the bilateral cerebellar hemispheres without accompanying parenchymal change (Figure 1). Her tamoxifen was switched to anastrozole. She underwent lumbar puncture for cerebrospinal fluid (CSF) analysis. This showed low glucose, elevated protein, and monocyte-predominant leukocytosis. Malignant cells consistent with adenocarcinoma were seen on cytology.

On Day 7 of admission, she became acutely disoriented, confused, and persistently febrile. Medications that could contribute to this altered mental status were stopped. Intravenous antibiotics were started empirically for the possibility of meningitis/cerebritis. Her labs showed leukocytosis with a negative infectious disease workup, which included bacterial and atypical/fungal infections (tuberculosis, West Nile, *Cryptococcus*, and histoplasma) due to the finding of low CSF glucose. Coombs-negative hemolysis with thrombocytopenia and elevated ferritin was present. Peripheral blood smear showed immature leukocytes (metamyelocytes and myelocytes), giant platelets, nucleated red blood cells, schistocytes, and teardrop cells.

**Figure 2:**
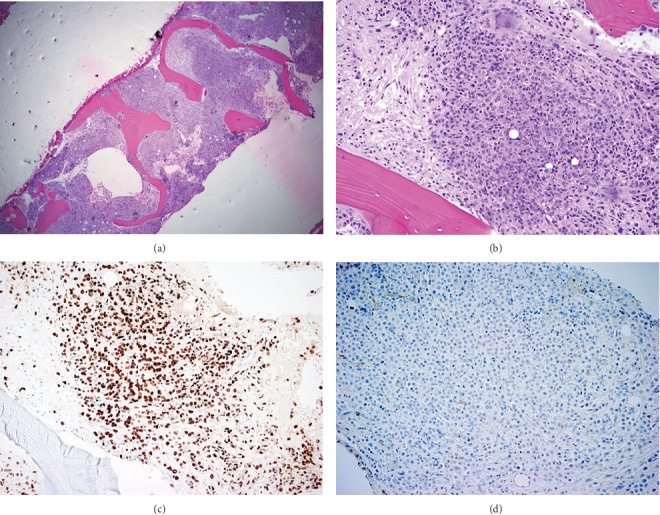
(a) Low magnification (4X) of the core biopsy shows near complete intramedullary replacement by metastatic carcinoma and absent trilineage hematopoiesis. (b) Medium magnification (20X) of the intertrabecular space shows complete replacement by metastatic carcinoma. (c) Immunohistochemical stain for GATA3 (20X) shows diffuse strong nuclear positivity, supportive of breast as the primary site of origin. (d) E-Cadherin (20X) shows negative staining in the carcinoma cells, demonstrating the lobular phenotype.

Due to an acute drop in hemoglobin of > 2.0 g/dL without obvious bleeding, a repeat anemia workup was undertaken (Table 2), with concern for hemophagocytic lymphohistiocytosis (HLH). Other differentials considered for this clinical picture were disseminated intravascular coagulation, thrombotic thrombocytopenic purpura, and tumor-associated thrombotic microangiopathy, which further workup did not support. A bone marrow biopsy was obtained for evaluation and showed extensive intramedullary marrow replacement by metastatic lobular breast carcinoma with associated diffuse reticulin fibrosis and absent trilineage hematopoiesis (Figure 2).

The patient started chemotherapy with weekly paclitaxel and intrathecal methotrexate. She followed up with the oncology clinic, where she continued to receive intrathecal methotrexate, with paclitaxel being discontinued due to toxicity. After 7 weeks, CSF analysis showed few atypical cells suspicious for malignancy. She started trastuzumab deruxtecan as second-line systemic therapy in light of blood–brain barrier (BBB) penetrance.

## 3. Discussion

ILC is the second most common type of breast cancer, with a higher median age at initial diagnosis as well as greater TNM stage at diagnosis [1]. ILC has a high propensity for metastases to bone (most common site), lung, peritoneum, gastrointestinal system, and gynecological organs [2]. The spread to bone is partly due to the expression of bone sialoprotein, a bone matrix protein thought to increase breast cancer cell affinity for bone [3]. ILC uniquely has a propensity for leptomeningeal spread, with prominent migration routes including hematogenous spread, adjacent parenchymal spread, and abluminal spread via bone marrow and dural vascular channels [4].

Leptomeningeal carcinomatosis is the seeding of the pia and arachnoid mater by either intracranial or extracranial cancers that may present alone or with parenchymal involvement. This occurs in up to 5% of patients with solid tumors, mainly occurring with breast cancer, lung cancer, and melanoma [5]. Infiltration may occur at the basal cisterns, posterior fossa, or cauda equina. Presenting symptoms may be due to increased intracranial pressure and include headaches (including positional), nausea, ataxia, cognitive impairment, and cranial nerve (especially CN 6) palsies [6]. Local inflammation is also responsible for seizures and cognitive impairment. When spinal nerves are involved, weakness, numbness, and bladder/bowel dysfunction may be present.

This patient's prior MVA presented a confounding factor as these symptoms can be attributed to trauma. However, these delayed symptoms raised concern for a new etiology. The incidence of leptomeningeal carcinomatosis may be increasing due to advanced imaging and better control of systemic disease with therapies that do not cross the BBB or the blood–CSF barrier. Diagnosis is made with imaging and/or CSF cytology. T1-weighted MRI with contrast will reveal enhancements of the leptomeninges (Figure 1). However, MRI may not detect even cytology-proven leptomeningeal carcinomatosis. Cytology is useful when imaging is negative. It has low sensitivity on initial attempt, and repeat sampling is recommended if leptomeningeal carcinomatosis is still suspected.

Stereotactic brain radiation, involved-field radiation, or whole brain radiation therapy may be used for patients with good clinical features. Intrathecal chemotherapy with methotrexate, liposomal cytarabine (combined with systemic therapy) [7], or trastuzumab [8] is recommended. Systemic chemotherapy with BBB-penetrating agents methotrexate (high dose), capecitabine, temozolomide, lomustine, pemetrexed, and topotecan can be added for augmentation [9].

The diagnosis of leptomeningeal carcinomatosis occurs less than 10 years from the breast cancer diagnosis [10]. Prognosis from the time of leptomeningeal carcinomatosis diagnosis is less than 2 years, even with chemoradiation [11]. A good functional status, initiating intrathecal chemotherapy at the time of diagnosis, and absence of concomitant brain parenchymal findings (or no findings on imaging) at the time of diagnosis are positive prognostic factors [12].

Iron overload—as evidenced by elevated ferritin and transferrin saturation (Table 2)—can be from increased iron absorption or turnover from ineffective erythropoiesis. The former was unlikely because of the absence of characteristic liver, skin, or endocrine manifestations as well as the presence of anemia. Ineffective erythropoiesis is classically seen in myelophthisic anemia, a normocytic, hypoproliferative anemia that represents bone marrow failure due to replacement by an abnormal infiltrate. Patients with significant marrow infiltration by tumor cells have a markedly reduced heme synthesis response to erythropoietin and impaired erythropoiesis [13], leading to premature destruction of RBC precursors. Subsequent incorporation of released iron into macrophages yields the labs suggestive of iron overload [14].

Myelophthisic anemia may be caused by solid tumor metastases, granulomatous diseases, lipid storage disorders, or primary myelofibrosis in the fibrotic phase. The solid cancers most associated with myelophthisic anemia are prostate, breast, and lung cancers, although this is a rare manifestation of any of them. A leukoerythroblastic reaction—immature erythrocytes and left-shifted myeloid precursors—is usually seen on peripheral blood smear [15]. Hepatosplenomegaly may also be present due to accompanying extramedullary hematopoiesis. Supportive treatment with blood transfusions may be used, but primary treatment should be targeted toward the underlying condition.

## Figures and Tables

**Table 1:** Our patient's laboratory values on presentation showing direct hyperbilirubinemia, transaminitis, mild anemia, mild thrombocytopenia, and elevated cancer antigens.

**Laboratory**	**Test**	**Value**	**Reference range (units)** ^**a**^
Liver panel	Total bilirubin	2.2	0.2–1.2 mg/dL
	Direct bilirubin	0.5	0.0–0.2 mg/dL
	AST	76	0–40 U/L
	ALT	42	5–35 U/L
	Alk. phosphatase	130	20–120 U/L

Complete blood count	WBC	14.4	4.4–10.6 k/*μ*L
	Neutrophils	60	45.3%–74.5%
	Bands	4	0%–9%
	Lymphocytes	18	18.1%–43.2%
	Monocytes	5	4.0%–11.1%
	Eosinophils	1	0.4%–5.8%
	Basophils	1	0.2%–1.0%
	Atypical lymphocytes	1	—
	Metamyelocyte	6	—
	Myelocyte	4	—
	HGB	12.7	12.9–16.8 g/dL
	HCT	37.1	38.1%–49.0%
	PLT	144	161–369 k/*μ*L

Others	CA15-3	452	< 32 U/mL
	CA27-29	798	< 38 U/mL

Abbreviations: ALT, alanine aminotransferase; AST, aspartate transaminase; CA, cancer antigen; HCT, hematocrit; HGB, hemoglobin; PLT, platelets; WBC, white blood cells.

^a^Reflects the reference range for our hospital's assays.

**Table 2:** Results of initial anemia workup and repeat workup done after acute drop in hemoglobin.

**Laboratory**	**Initial**	**Repeat**	**Reference range (units)** ^**a**^
Iron	102	110	45–182 *μ*g/dL
UIBC	70.4	< 55	155–300 *μ*g/dL
TIBC	172	Incalculable	250–425 *μ*g/dL
% saturation	59.3	—	20%–50%
Ferritin	4243.58	6546.15	11.0–306.8 ng/mL
Reticulocyte index	—	1.6	

Abbreviations: TIBC, total iron binding capacity; UIBC, unsaturated iron binding capacity.

^a^Reflects the reference range for our hospital's assays.

## Data Availability

Data sharing is not applicable to this article as no datasets were generated or analyzed during the current study.
